# Exploring the Dietary Strategies of Coated Sodium Butyrate: Improving Antioxidant Capacity, Meat Quality, Fatty Acid Composition, and Gut Health in Broilers

**DOI:** 10.3390/genes16040433

**Published:** 2025-04-05

**Authors:** Zhuoya Gu, Wenwu Xu, Tiantian Gu, Lizhi Lu, Guohong Chen

**Affiliations:** 1 Jiangsu Key Laboratory for Animal Genetic, Breeding and Molecular Design, Yangzhou University, Yangzhou 225009, China; zhuoya_gu@163.com; 2 Institute of Animal Husbandry and Veterinary Science, Zhejiang Academy of Agricultural Sciences, Hangzhou 310021, China; xuwenwu248@outlook.com (W.X.); gutiantian1029@outlook.com (T.G.)

**Keywords:** coated sodium butyrate, antioxidant capacity, meat quality, gut microbiota, broiler

## Abstract

Background/Objectives: Broiler chickens are excellent animals for protein production and play an essential role in the food industry. The purpose of this study is to investigate the effect of coated sodium butyrate (CSB) on the biochemical indices, antioxidant capacity, meat quality, fatty acid composition, and gut health of Xianju broilers. Methods: A total of 192 one-day-old broilers were randomly divided into four treatment groups: the basal diet (CK), the basal diet with 250 mg/kg CSB (CSB250), the basal diet with 500 mg/kg CSB500 (CSB500), and the basal diet with 1000 mg/kg CSB (CSB1000). Each group included six replicates, with eight chicks per replicate. Results: We found that CSB supplementation in the diets has no function on plasma biochemical indices; however, CSB1000 broilers exhibited markedly elevated plasma TG levels. Furthermore, CSB supplementation at different concentrations significantly increased plasma antioxidase capacity in broilers. Moreover, breast meat supplemented with CSB displayed a higher shear force, pH_24h_, and inosinic acid content than CK meat. Breast meat of broilers fed CSB1000 showed improved fatty acid composition, evidenced by increased levels of polyunsaturated fatty acids (C16:1, C18:2, C22:4, and C22:6). Moreover, supplementation with CSB1000 optimized the gut microbiota composition, particularly by enhancing the abundance of Firmicutes and the Firmicutes/Bacteroidetes ratio. Conclusions: Collectively, these findings offer a basis for the extensive application of CSB as a feed addition to enhance the quality of meat in the broiler sector.

## 1. Introduction

Broilers are among the most efficient animals for protein production and play an essential role in human growth and development worldwide [1]. The consumption of poultry products has become more popular due to the high nutritional value, rich essential amino acid levels, low fat content, and high digestibility of broiler meat [2]. To meet consumer demand, the industry employs intensive manual selection and high-energy dietary inputs to increase carcass yield [3,4,5]. Nevertheless, due to the intensive selection of production traits, broilers present several challenges, leading to poor meat quality [6]. Studies have emphasized that feed supplementation, a crucial connection between animal production, food technology, and human nutrition, plays a role in changing meat quality [7]. Therefore, it is necessary to develop effective dietary strategies to improve meat quality.

Butyrate, a short-chain fatty acid, is primarily generated through the microbial fermentation of indigestible carbohydrates in the diet [8]. Evidence has shown that sodium butyrate (SB) can be absorbed in the intestine of pigs and birds by transforming butyric acid in the tract [9,10,11,12]. In addition to its adsorption properties, SB is a safe and environmentally acceptable feed additive for animal production that can improve poultry growth performance and intestinal health [13]. However, owing to the low pH of the intestine, the bioavailability of butyrate is limited to the hindgut [14]. Therefore, it is appropriate to produce physically and chemically coated butyrates. Recent studies have found that coated sodium butyrate (CSB) strengthens growth performance and immunity through gut microbiota alteration in poultry [15,16].

The gut microbiota is believed to influence many metabolic processes, such as nutrient absorption, host health, and meat quality [17,18,19]. Studies have focused on the relationship between the gut microbiome altered by feed supplementation and meat quality [20,21]. In chickens, the feed additive of tea polysaccharide enriched the microbial diversity and abundance of Bacteroidetes and Lactobacillus, which in turn improved the pH and redness (a*) value of breast meat [22]. Dietary administration of Anoectochilus roxburghii extract can reduce abdominal fat deposition and improve meat quality by modulating the ratio of Firmicutes to Bacteroidetes [23]. Many studies have reported that CSB can improve the gut microbiota composition in poultry [16,23]; however, the role of the CSB—induced microbiota in improving meat quality has not been fully investigated.

Therefore, this study sought to determine the changes in antioxidant capacity, meat quality, fatty acid composition, and gut microbiota composition following the addition of CSB to broiler diets.

## 2. Materials and Methods

### 2.1. Ethics Statement

Animal use and care protocols were approved by the Animal Use and Care Committee of Yangzhou University, Yangzhou, China (Approval code: YZUDWSY2022-279, Approval date: 6 August 2022).

### 2.2. Experimental Design and Sample Collection

Xianju broilers and CSB with intelligent microcapsule encapsulation were purchased from the Zhejiang Xianju chicken breeding farm (Tiantai, Zhejiang, China) and Zhejiang King Technology Co., Ltd. (Hangzhou, Zhejiang, China), respectively. One hundred and ninety-two one-day-old healthy Xianju broilers were randomly divided into four groups: CK (the basal diet), CSB250 (the basal diet with 250 mg/kg CSB), CSB500 (the basal diet with 500 mg/kg CSB), and CSB1000 (the basal diet with 1000 mg/kg CSB). Each group included six replicates with eight broilers per replicate, and the experimental period lasted ten weeks. At the end of the trial period, one broiler was randomly selected from each replicate for subsequent analysis, yielding six biological replicates per group for sample collection and measurements. Dietary nutrient levels were based on the National Research Council’s recommended nutrient requirements for broiler chickens (Table 1). The broilers were maintained in three-tiered cages (100 × 45 × 70 cm per tier), each provisioned with ad libitum access to feed and water. Environmental conditions were strictly regulated, with an initial temperature of 33 °C during the first 72 h, followed by a systematic reduction of 1 °C every 48 h until reaching a stable 24 °C, which was sustained throughout the remaining experimental period.

After ten weeks, blood samples were obtained from the subcutaneous vein. All samples were centrifuged at 3000 rpm for 10 min at 4 °C to obtain plasma and then removed into −80 °C for further analysis. The broilers were euthanized by intravenous injection of sodium pentobarbital [200 mg/kg body weight (BW)]. Breast muscle and cecal samples were collected, immediately snap-frozen in liquid nitrogen, and stored at −80 °C for nutritional profile and 16s rRNA analyses, respectively. A portion of breast muscles was stored at 4 °C for meat quality measurement.

### 2.3. Plasma Biochemical Indexes

Plasma biochemical indicators, including the total protein (TP), albumin (ALB), globulin (GLB), aspartate aminotransferase (AST), alanine aminotransferase (ALT), total cholesterol (TC), triglycerides (TG), high density lipoprotein (HDL), and low density lipoprotein (LDL), were measured according to the manufacturer’s instructions (Sinoukbio, Co., Ltd., Beijing, China).

### 2.4. Oxidative Stress Biomarkers

The total antioxidant capacity (T-AOC), catalase (CAT), superoxide dismutase (SOD), and glutathione peroxidase (GSH-PX) activity, and malondialdehyde (MDA) content in the plasma were determined according to the manufacturer’s instructions (Solarbio, Beijing, China).

### 2.5. Meat Quality Traits

After broilers were slaughtered, breast muscles were immediately used to detect the meat color and shear force. The meat color, including L* (lightness), a* (redness), and b* (yellowness), was evaluated using a portable colorimeter (Chroma Meter CR-410; Konica Minolta, Tokyo, Japan). The shear force was examined with a Warner-Bratzkr Meat Shear (G-R151, G-R Co., Minneapolis, MN, USA). Then, 24 h after slaughter, the pH_24h_ analysis was performed with a portable meat pH meter (pH-STAR, MATTHAUS, Essen, Germany) at a depth of 10 mm into the breast meat overnight.

### 2.6. Inosine 5′-Monophosphate (IMP)

Breast muscle samples were mixed with 10 mL of 10% perchloric acid in a glass tube, crushed for 30 min using an ultrasonic processor, and centrifuged at 4000× *g* for 15 min to obtain the supernatant. Subsequently, a NaOH solution was added to adjust the pH to 6.5. Finally, the solution was filtered through a 0.22 μm membrane filter into an autosampler, and IMP content was detected under Agilent C18 (4.6 mm × 250 mm × 5 μm; Agilent, Santa Clara, CA, USA).

### 2.7. Free Fatty Acid (FFA) Analysis

Free fatty acid analysis of breast meat was performed using gas chromatography, as described by Fan et al. [24]. Briefly, 20 g of the breast sample was weighed, dried, ground, and immersed in a chloroform-methanol mixture (2:1) for extraction. Subsequently, KOH-CH_3_OH was added for saponification, followed by the addition of a BF_3_-CH_3_OH solution, and then was thoroughly mixed. The mixture was then subjected to a 20 min incubation in a water bath at 85 °C. Finally, hexane and NaCl solutions were added for extraction, and fatty acid methyl esters were determined using a 7890A instrument (Agilent, CA, USA). The gas chromatograph was conducted using a split injection mode (10:1) with a 1.0 μL sample volume. High-purity helium carrier gas was delivered at a constant flow rate of 1.0 mL/min. The inlet and detector temperatures were maintained at 270 °C and 280 °C, respectively. The oven temperature program was initiated at 100 °C (isothermal for 13 min), followed by a gradient increase to 180 °C at 10 °C/min (6 min hold), then a slower ramp to 200 °C at 1 °C/min (20 min hold), and finally a rise to 230 °C at 4 °C/min (10.5 min hold). Fatty acid methyl esters (FAMEs) were identified by comparing their retention indices to those of authenticated reference standards. The results are expressed as g/kg of total identified fatty acids.

### 2.8. Intramuscular Fat

The breast muscles were cut into pieces and ground into a paste using a high-speed universal crusher. Approximately 180 g of ground muscle was placed in a 140 mm round sample cup to determine the intramuscular fat content using a FoodScan™ Meat Analyzer (Foss, Hilleroed, Denmark).

### 2.9. 16s rRNA Sequencing

Total genomic DNA was extracted using a DNA isolation kit (M5635-02; Omega, Norcross, GA, USA). The obtained DNA was amplified using the primer designed on the V3–V4 regions (F: ACTCCTACGGGAGGCAGCA; R: GGACTACHVGGGTWTCTAAT). Sequencing libraries were constructed and performed by Personalbio Technology (Shanghai, China). Subsequently, the amplicon sequencing was performed using the Illumina MiSeqTM System (Illumina, San Diego, CA, USA). The sequencing data were then used to determine the amplicon sequence variants (ASVs) for diversity analysis as well as for statistical analysis using the QIIME (2.0) software. To characterize the gut microbial community dynamics, we computed multiple α—diversity metrics and performed principal coordinate analysis (PCoA) based on β diversity using QIIME 2. Additionally, the linear discriminant analysis effect size (LEfSe) was applied to identify differentially abundant taxa across study groups.

### 2.10. Statistical Analysis

Results are expressed as means ± standard deviations (SDs). Significance tests were performed using SPSS 25.0, with one-way ANOVA. *p* < 0.05 was the threshold for statistical significance. GraphPad Prism (version 8.0) was used to create the graphs.

## 3. Results

### 3.1. Biochemical Parameters

As shown in Table 1, dietary CSB supplementation did not alter the levels of plasma TP, ALB, GLB, AST, ALT, TC, HDL, or LDL (*p* > 0.05). Dietary supplementation with different concentrations of CSB increased TG content. The TG content in the CSB1000 group was noticeably increased compared to the CK group (*p* < 0.05, Table 2).

### 3.2. Antioxidant Capacity

**Figure 1:**
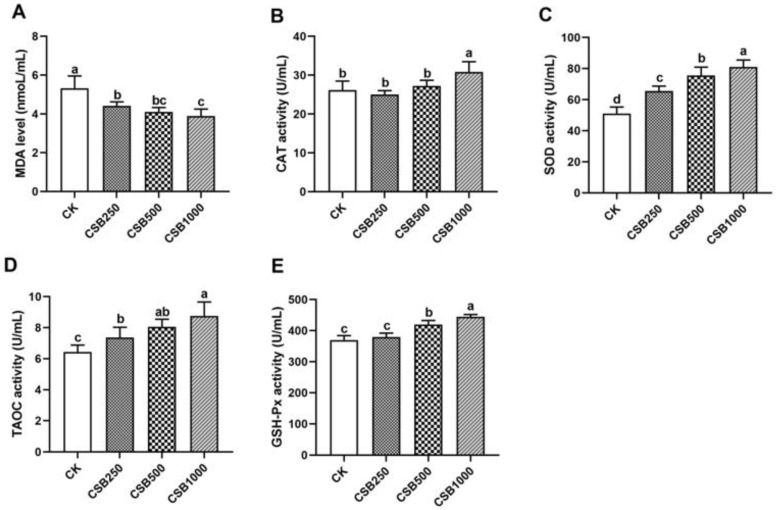
Effects of CSB supplementation on antioxidant capacity in broilers. (**A**) MDA level. (**B**) CAT activity. (**C**) SOD activity. (**D**) T-AOC activity. (**E**) GSH-Px activity. *n* = 6 for each group. ^a–d^ Means with different letters indicate statistically significant differences (*p* < 0.05).

The plasma antioxidant indices after dietary CSB supplementation are presented in Figure 1. MDA levels showed a marked decreasing trend with different concentrations of CSB supplementation (*p* < 0.05; Figure 1A). Significantly higher CAT activity was observed in broilers fed diets supplemented with 1000 mg/kg CSB compared to broilers in the CK group (*p* < 0.05, Figure 1B), whereas CAT activity in broilers fed diets supplemented with 250 and 500 mg/kg CSB showed no significant difference compared to the CK group. Additionally, SOD and T-AOC activities in the different CSB concentration groups indicated a markedly increasing tendency compared to the CK group (*p* < 0.05, Figure 1C,D). GSH-Px activity in the diets supplemented with different concentrations of CSB (except for CSB250 group) was noticeably higher than in the CK group (*p* < 0.05, Figure 1E).

### 3.3. Meat Quality

**Figure 2:**
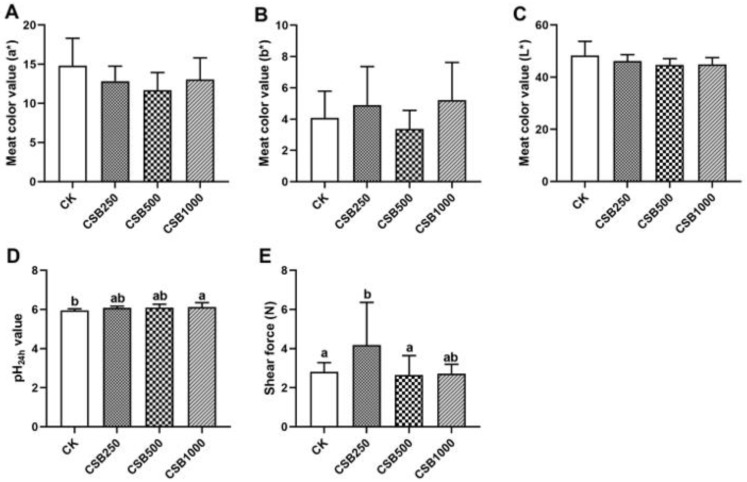
Effects of CSB supplementation on meat quality in broilers. (**A**) Meat color value (a*). (**B**) Meat color value (b*). (**C**) Meat color value (L*). (**D**) pH_24h_ value. (**E**) Shear force. *n* = 6 for each group. ^a,b^ Means with different letters indicate statistically significant differences (*p* < 0.05).

To determine whether dietary CSB supplementation could improve meat quality in broilers, the representative physical properties were investigated (Figure 2). The breast muscle of broilers that received CSB supplementation showed no significant change in meat color (a*, b*, and L*) (*p* > 0.05, Figure 2A–C). In addition, higher pH_24h_ values in the breast muscle were observed in the CSB1000 group compared to those in the CK group (*p* < 0.05, Figure 2D), and a higher shear force was detected in the CSB250 group compared to the CK group (*p* < 0.05, Figure 2E).

### 3.4. Inosinic Acid and Intramuscular Fat Content

We compared the inosinic acid and intramuscular fat contents under the influence of CSB. As shown in Table 3, the IMP content of breast muscle in broilers fed 500 and 1000 mg/kg CSB markedly increased compared to the CK group (*p* < 0.05). However, the intramuscular fat content was not significantly different with or without CSB (*p* > 0.05).

### 3.5. Free Fatty Acid Composition

Nine FFAs were detected in all meat samples: three saturated fatty acids (SFAs), two monounsaturated fatty acids (MUFAs), and four polyunsaturated fatty acids (PUFAs) (Table 4). Dietary CSB supplementation markedly increased C16:1, C18:2, C22:4, and C22:6 levels (*p* < 0.05). However, C18:0 content showed a slight but significant reduction in the CSB groups compared to the CK group (*p* < 0.05). CSB treatments had no influence on SFA or MUFA levels (*p* > 0.05); however, the PUFA contents in the CSB-supplemented groups (except for the CSB250 group) were significantly reduced compared to the CK group.

### 3.6. Gut Microbiota Compositions

**Figure 3:**
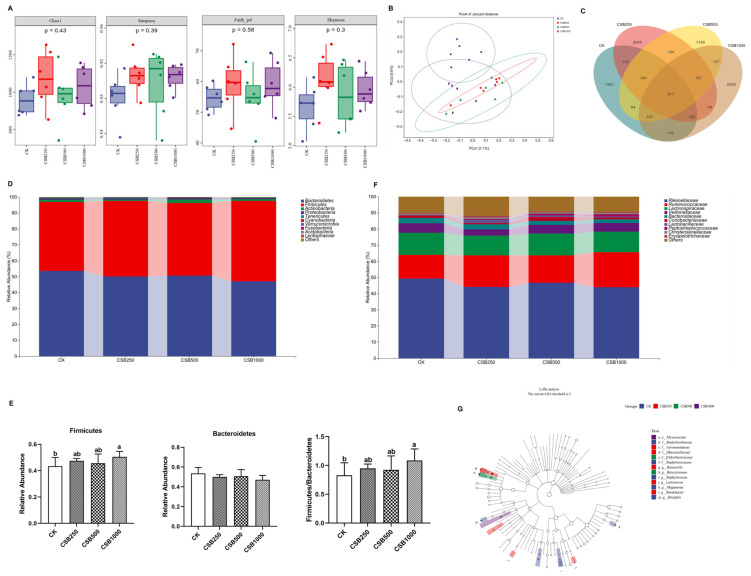
Effects of CSB supplementation on the gut microbiota compositions in broilers. (**A**) Variations in α diversity. (**B**) PCoA analysis. (**C**) Venn diagram summarizing the numbers of ASVs in the cecal microorganisms. (**D**) Microbiota composition analysis at the phylum level. (**E**) Relative abundance of Firmicutes and Bacteroidetes. (**F**) Microbiota composition analysis at the family level. (**G**) LEfSe analysis. *n* = 6 for each group. ^a,b^ Means with different letters indicate statistically significant differences (*p* < 0.05).

To further examine the role of CSB in regulating the gut microbiota, 16S rRNA sequencing was performed. A total of 24,455,609 high-quality clean reads were generated from 24 cecal samples. The α diversity (represented by the Chao1, Simpson, Faith_pd, and Shannon indices) of the intestinal flora showed no significant difference with the CSB addition compared to the CK group (*p* > 0.05, Figure 3A). PCoA analysis indicated that the microbiomes from the CK and CSB supplementation groups were in distinct clusters (Figure 3B). A Venn diagram showed that 817 amplicon sequence variants (ASVs) of the microbiota were obtained among the CK, CSB250, CSB500, and CSB1000 groups (1801, 2049, 1780, and 2085 unique ASVs, respectively) (Figure 3C). As shown in Figure 3D, Firmicutes and Bacteroidetes were the most abundant phyla. The relative abundance of Firmicutes and the ratio of Firmicutes/Bacteroidetes in CSB—supplemented broilers were significantly increased (*p* < 0.05, Figure 3E). Furthermore, Rikenellaceae, Ruminococcaceae, and Lachnospiraceae were the dominant species (Figure 3F). LEfSe analysis showed that the genera Staphylococcus, Megamonas, and Bilophila in the CK group, Barnesiella, Lactococcus, and Rhodobacter in the CSB250 group, and Butyricimonas in the CSB500 group were the predominant bacterial strains (Figure 3G).

## 4. Discussion

The utilization of CSB to boost broiler productivity and overall health has become more popular because of its biological properties, which include hypoglycemic advantages and broad enhancements in growth performance, antioxidant capacity, immune function, and intestinal health [24]. These findings highlight that dietary incorporation of CSB improves gut health, microbiota, antioxidant capacity, and immunity in poultry [16,25,26]. However, few studies have been conducted on how adding CSB to the diet can improve meat quality through the regulation of the gut microbiota in broiler chickens.

Animal physiological and metabolic states can be inferred from serum indices, which are influenced by a variety of environmental conditions [27]. TP indicates the existence of chronic inflammatory or infectious disorders, as well as the body’s nutritional state and liver and kidney function [28,29]. A part of TP, GLB is essential for both protein transport and the immunological response. Low GLB levels imply malnourishment or problems with protein synthesis, while elevated GLB levels may be a sign of immunological diseases, liver illness, or chronic inflammatory conditions [30,31,32]. Furthermore, AST and ALT are amino acid transferases that are important biomarkers of liver function [33]. In the present study, no significant differences were observed in the levels of TP, ALB, GLB, AST, and ALT, indicating that CSB supplementation had no effect on broiler body function.

The generation of reactive oxygen species (ROS) surpasses the ability of cellular antioxidant defenses to eliminate these harmful species, resulting in oxidative stress. Antioxidants have the potential to inhibit ROS generation or scavenge free radicals. Previous studies have emphasized the positive effects of dietary supplementation with butyric acid, which is mainly associated with antioxidant capacity and improved chicken immune response [34,35]. Dietary CSB increased serum SOD and T-AOC activities and decreased MDA content in laying ducks [16]. Moreover, it was reported that a basal diet supplemented with CSB fed to laying hens not only enhanced the activity of GSH-Px but also decreased MDA levels, a lipid peroxidation marker, in the serum and liver [35]. In the current study, significant increases in SOD, CAT, and T-AOC activities, along with a reduction in MDA content, were observed in the plasma of CSB-fed broilers. Collectively, our findings revealed that broilers fed diets supplemented with CSB exhibited a strong antioxidant capacity.

Meat color is the most important factor affecting the likelihood of consumers purchasing livestock and poultry meat products. However, the present study showed that the addition of CSB had no significant effect on breast muscle color. Further, shear force is an objective index that evaluates meat tenderness [36], and dietary CSB resulted in increased shear force on the breast muscles in the present study, indicating reduced tenderness. Consequently, dietary supplementation with acidifiers may be required to improve meat tenderness in broiler chickens fed with CBS-supplemented diets. Additionally, pH is a crucial indicator of meat quality [37], and the pH_24h_ values of CSB-treated broilers in breast muscles were significantly higher than those of CK broilers, indicating that dietary CSB supplementation could improve meat quality by inhibiting the reduction in pH values. Meat pH levels are strongly influenced by oxidative stress and antioxidant capacity [38]. For instance, supplementing diets with Dendrobium officinale leaves (DOLs) has been shown to elevate broiler meat pH, likely attributable to DOL’s ability to enhance antioxidant enzyme activity [37]. Other authors have reported that meat quality is directly correlated with the antioxidant state of the body before slaughter [39]. Our results demonstrate that dietary CSB is effective in promoting antioxidant capacity and improving meat quality.

FFA composition plays a major role in meat quality traits, including sensory properties and nutritional values [40]. Human nutritionists recommend a high intake of PUFAs, which have a wide range of biological roles and are believed to be beneficial to human health [41,42]. In the present study, dietary supplementation of broilers with CSB increased the PUFA content, especially C18:2, C22:4, and C22:6. This may be because the antioxidant-active ingredients in CSB prevented PUFA oxidation and decreased the oxidation of fatty acids in breast muscle, which has also been indicated in other feed additives [43]. In addition to FFA, inosine 5′-monophosphate (IMP) is an endogenous compound in muscle and is known to contribute to meat flavor and umami taste [44]. We observed that CSB significantly influenced IMP content, which was higher in breast meat from CSB broilers than that from CK broilers. Considering the higher IMP content and a more favorable balance between n-6 and n-3 PUFAs, broiler meat supplemented with CSB is more beneficial for consumer health.

The intestinal microflora is a complex ecosystem composed of many microorganisms that play important roles in maintaining the intestinal function and health of the host. Our results indicated that CSB treatment enhanced the relative abundance of Firmicutes and the ratio of Firmicutes/Bacteroidetes, as well as improving meat performance [22]. Yang et al. suggested that an increase in the abundance of Firmicutes has been linked to improved lipid accumulation and meat flavor [45]. Thus, CSB may provide a positive strategy for modulating the gut microbial community and improving the meat quality of broiler chickens, which can be applicable for poultry farmers. However, the limitation of this study is the effect of CSB in the relationship between the intestinal microbial composition and meat quality, and future studies need to address mechanistic links through targeted metabolomics (e.g., SCFA quantification) and functional metagenomics.

## 5. Conclusions

This study provides novel evidence indicating the potential of CSB as a feed supplement for enhancing broiler meat quality. The inclusion of CSB in broiler diets showed potent antioxidant activity, positively influencing broiler meat quality, fatty acid composition, and gut microbial community composition. However, more investigation is required to fully comprehend the mechanisms at play and the ideal levels of CSB incorporation for broiler productivity.

## Figures and Tables

**Table 1:** The composition and nutrient levels of the experimental diets.

Items	1~42 d	43~70 d
Ingredient (%)		
Corn	53.7	35.6
Soybean meal	23	8.2
Extruded soybean	6	2
Rice bran	6.5	6
Soybean oil	0.8	1.4
Corn gluten meal	3	4
Limestone	1.33	1.3
Fermented soybean meal	1.67	
Wheat grain		18
Rice bran		6
DDGS (corn) ^1^		10
Wheat red dog		0.3
Premix ^2^	4	3.2
Total	100	100
Nutrient composition, calculated		
Metabolizable energy (Kcal/kg)	2950	2997
Crude protein (%)	21.1	16.7
Crude fat (%)	4.8	5.5
Calcium (%)	0.87	0.70
Total phosphorus (%)	0.63	0.58
Lysine (%)	1.22	0.95
Tryptophan (%)	0.22	0.19
Methionine (%)	0.54	0.40
Threonine (%)	0.85	0.67
Methionine and cysteine (%)	0.88	0.72
Analyzed nutrient components		
Crude protein	21.12	16.34
Crude fat	4.89	5.58
Crude ash	5.04	5.53
Dry matter	89.75	90.24

^1^ DDGS, Distillers’ dried grains with solubles. ^2^ Premix provided per kilogram of diet: vitamin A, 1000 IU; vitamin D3, 250 IU; vitamin E (DL-α-tocopheryl acetate), 15 mg; vitamin B1, 3.6 mg; vitamin B2, 2.8 mg; vitamin B6, 4.1 mg; Cu (as CuSO_4_·5H_2_O), 7.5 mg; Fe (as FeSO_4_·7H_2_O), 75 mg; Zn (as ZnSO_4_), 51.75 mg; Mn (as MnSO_4_), 55.65 mg; I (as Ca(IO_3_)_2_), 0.1 mg; and Se (as NaSeO_3_·5H_2_O), 0.05 mg.

**Table 2:** Effects of CSB supplementation on biochemical parameters in broilers.

Items	CK	CSB250	CSB500	CSB1000
TP (g/L)	10.92 ± 2.96	13.60 ± 10.57	9.05 ± 3.13	15.17 ± 9.05
ALB (g/L)	5.94 ± 1.57	7.68 ± 6.23	4.97 ± 2.10	9.14 ± 6.63
GLB (g/L)	4.98 ± 1.44	5.93 ± 4.37	4.08 ± 1.06	6.03 ± 2.54
AST (U/L)	102.05 ± 32.77	100.50 ± 76.15	66.80 ± 19.98	106.74 ± 49.36
ALT (U/L)	5.61 ± 0.42	5.82 ± 1.01	4.89 ± 0.68	5.37 ± 0.74
TC (mmol/L)	1.19 ± 0.31	1.43 ± 0.98	1.10 ± 0.47	1.77 ± 0.94
TG (mmol/L)	0.10 ± 0.03 ^b^	0.16 ± 0.09 ^ab^	0.14 ± 0.04 ^ab^	0.20 ± 0.12 ^a^
HDL (mmol/L)	0.70 ± 0.18	0.83 ± 0.49	0.62 ± 0.27	0.91 ± 0.36
LDL (mmol/L)	0.35 ± 0.14	0.37 ± 0.32	0.27 ± 0.14	0.54 ± 0.41

CSB, coated sodium butyrate; TP, total protein; ALB, albumin; GLB, globulin; AST, aspartate aminotransferase; ALT, alanine aminotransferase; TC, total cholesterol; TG, triglyceride; HDL, high-density lipoprotein; LDL, low-density lipoprotein. *n* = 6 for each group. ^a,b^ Means with different letters are significantly different (*p* < 0.05).

**Table 3:** Effects of CSB supplementation on inosinic acid and intramuscular fat in broilers.

Items	CK	CSB250	CSB500	CSB1000
IMP (mg/g)	1.33 ± 0.11 ^b^	1.42 ± 0.06 ^ab^	1.44 ± 0.09 ^a^	1.45 ± 0.09 ^a^
Intramuscular fat (%)	1.97 ± 0.13	2.01 ± 0.08	2.11 ± 0.11	2.04 ± 0.15

IMP, inosine 5′-monophosphate. *n* = 6 for each group. ^a,b^ Means with different letters are significantly different (*p* < 0.05).

**Table 4:** Effects of CSB supplementation on free fatty acid compositions in broilers.

FFA (g/kg)	CK	CSB250	CSB500	CSB1000
C14:0	4.03 ± 0.13	3.98 ± 0.09	3.93 ± 0.09	3.93 ± 0.04
C16:0	199.89 ± 2.88	198.89 ± 4.34	201.83 ± 6.06	201.42 ± 7.04
C16:1	24.74 ± 0.57 ^b^	25.62 ± 0.70 ^a^	24.34 ± 0.52 ^b^	24.36 ± 0.43 ^b^
C18:0	89.10 ± 1.25 ^a^	89.56 ± 1.08 ^a^	86.36 ± 1.92 ^b^	87.37 ± 0.65 ^b^
C18:1	259.06 ± 1.64	260.56 ± 2.75	260.98 ± 4.30	261.28 ± 4.01
C18:2	163.83 ± 1.44 ^b^	165.06 ± 2.37 ^ab^	166.25 ± 1.21 ^a^	165.99 ± 1.80 ^a^
C18:3	8.33 ± 0.11	8.45 ± 0.12	8.34 ± 0.08	8.40 ± 0.17
C22:4	11.84 ± 0.24 ^b^	12.07 ± 0.07 ^a^	12.21 ± 0.12 ^a^	12.18 ± 0.16 ^a^
C22:6	14.12 ± 0.15 ^b^	14.22 ± 0.08 ^ab^	14.34 ± 0.22 ^a^	14.32 ± 0.06 ^a^
SFA	293.02 ± 2.80	292.42 ± 3.56	292.12 ± 7.14	292.72 ± 7.18
MUFA	283.80 ± 1.57	286.18 ± 2.31	285.32 ± 4.42	285.64 ± 3.70
PUFA	198.11 ± 1.36 ^b^	199.79 ± 2.44 ^ab^	201.13 ± 1.34 ^a^	200.88 ± 1.98 ^a^

FFA, free fatty acid; SFA, saturated fatty acid = C14:0 + C16:0 + C18:0; MUFA, monounsaturated fatty acid = C16:1 + C18:1; PUFA, polyunsaturated fatty acid = C18:2 + C18:3 + C22:4 + C22:6. *n* = 6 for each group. ^a,b^ Means with different letters are significantly different (*p* < 0.05).

## Data Availability

The 16S rRNA sequencing read data generated in this study have been uploaded to the Genome Sequence Archive database with the accession number CRA024100.
